# Nuclear Localization of the DNA Repair Scaffold XRCC1: Uncovering the Functional Role of a Bipartite NLS

**DOI:** 10.1038/srep13405

**Published:** 2015-08-25

**Authors:** Thomas W. Kirby, Natalie R. Gassman, Cassandra E. Smith, Lars C. Pedersen, Scott A. Gabel, Mack Sobhany, Samuel H. Wilson, Robert E. London

**Affiliations:** 1 Genome Integrity & Structural Biology Laboratory, NIEHS, National Institutes of Health, Research Triangle Park, North Carolina 27709.

## Abstract

We have characterized the nuclear localization signal (NLS) of XRCC1 structurally using X-ray crystallography and functionally using fluorescence imaging. Crystallography and binding studies confirm the bipartite nature of the XRCC1 NLS interaction with Importin α (Impα) in which the major and minor binding motifs are separated by >20 residues, and resolve previous inconsistent determinations. Binding studies of peptides corresponding to the bipartite NLS, as well as its major and minor binding motifs, to both wild-type and mutated forms of Impα reveal pronounced cooperative binding behavior that is generated by the proximity effect of the tethered major and minor motifs of the NLS. The cooperativity stems from the increased local concentration of the second motif near its cognate binding site that is a consequence of the stepwise binding behavior of the bipartite NLS. We predict that the stepwise dissociation of the NLS from Impα facilitates unloading by providing a partially complexed intermediate that is available for competitive binding by Nup50 or the Importin β binding domain. This behavior provides a basis for meeting the intrinsically conflicting high affinity and high flux requirements of an efficient nuclear transport system.

The localization, complexity and variability of DNA damage requires a complex cellular response that often utilizes scaffold proteins to recruit and coordinate the activities of the individual repair enzymes required to correct the damage. The X-ray cross complementing group 1 protein (XRCC1) is a scaffold that plays important roles in the overlapping single strand break repair (SSBR) and base excision repair (BER) pathways^1^,^2^,^3^,^4^, and participates in other repair pathways as well^5^,^6^. In order to coordinate DNA repair events, XRCC1 must be targeted to the nuclear compartment and to sites of DNA damage. Damage targeting of XRCC1 is multifaceted, but one mechanism utilizes a poly(ADP-ribose) (PAR)-dependent recruitment system involving PAR binding by the XRCC1 BRCTa domain^7^,^8^,^9^. In addition, PAR-independent mechanisms are also involved^10^,^11^,^12^. The nuclear uptake of XRCC1 is believed to utilize the classical nuclear transport system, however, information about nuclear uptake has been limited and inconsistent.

Nuclear import is directed through nuclear localization sequences (NLSs). Classical nuclear localization sequences are either monopartite, consisting of a single cluster of basic residues, or bipartite, consisting of two neighboring clusters of basic residues^13^. These sequences are recognized by the protein Importin α (Impα), which binds to the NLS of the cargo protein in the cytoplasm, and then translocates it along with Importin β through the nuclear pore complex into the nucleus^14^. A bipartite NLS corresponding to XRCC1 residues 239–266 was initially identified by Masson *et al.*, who demonstrated that this sequence could mediate the nuclear uptake of β-galactosidase^8^. A subsequent study by Kiriyama *et al.* fused several variations of the XRCC1 NLS to the repair protein aprataxin in order to effect nuclear uptake. These studies indicated that the minimum segment required for efficient nuclear transport included an additional 10 residues, 239–276^15^. Computational prediction of potential XRCC1 NLS motifs^16^ resulted in the identification of two monopartite sequences corresponding to residues 242–250 and 267–276. These two regions are included in the extended NLS identified by Kiriyama *et al.*^15^, however, the separation between the two binding motifs is atypically long (Fig. 1). Hence, there is currently some ambiguity regarding the identification and properties of the XRCC1 NLS. Nevertheless, it has become increasingly clear that bipartite NLS signals often include linkers that significantly exceed the 10–12 residues initially identified in such signals^13^,^17^,^18^,^19^,^20^,^21^,^22^,^23^,^24^.

XRCC1 represents a challenging cargo protein for the nuclear import machinery due to its floppy structure that includes two unstructured linker sequences greater than 120 residues in length, as well as its ability to interact with multiple DNA-repair binding partners. Although for most of these binding partners a role of XRCC1 in nuclear uptake is unclear, XRCC1 is apparently required for the co-transport of constitutively bound DNA ligase 3α (Lig3α). Alternative splicing of the Lig3 gene results in both mitochondrial and nuclear forms of Lig3α and germ cell-specific nuclear Lig3β^25^,^26^. Sequence analysis indicates that Lig3β contains a C-terminal NLS, while Lig3α contains a C-terminal BRCT domain that mediates binding to XRCC1 but lacks an NLS^27^,^28^. Thus, for the α-form, nuclear uptake is dependent on XRCC1 co-transport. More recently, XRCC1 also has been shown to mediate nuclear translocation of JWA, a microtubule-associated protein involved in activation of MAPK cascades^29^.

In view of these variable results for the XRCC1 NLS and the essential requirement of nuclear localization of XRCC1 in order to fulfill its repair functions as a scaffold protein, we have further investigated the structural basis for the nuclear uptake of this protein. Detailed binding studies of the bipartite NLS peptide, as well as its separate major and minor binding motifs, to both wild-type and mutated forms of Impα reveal pronounced cooperative binding behavior that is due to the tethering of the major and minor motifs. This behavior provides a basis for meeting the intrinsically conflicting high affinity and high flux requirements of an efficient nuclear transport system.

## Results

### Structural characterization of the XRCC1 NLS complex with Importin α

For crystallographic structural analysis of the complex formed between the hXRCC1 nuclear localization sequence and murine Impα(70–529), we selected a peptide that encompasses all of the previously proposed XRCC1 NLS binding motifs (amino acids 241–276). The expectation was that an observed structure would correspond to the most stable complex. It is well known that lattice contacts can influence crystallization, so the peptide length was a potential confounding factor. However, in reported crystal structures, NLS peptides bind to the concave interior surface of the Importin molecule and in general do not appear to be involved in lattice contacts. Hence, intermolecular lattice contact interactions do not appear to play a role in selecting the observed Importin-peptide complex. The Impα(70–529) was chosen because this construct, with the Importin β binding domain (IBB) deleted, is identical to that used in previous crystallography studies by Fontes *et al.*^30^.

The NLS-binding domain of Impα contains ten armadillo (arm) repeats that are comprised of three helices each, with the exceptions of: “arm 1” that, in our structure, starts just before the second helix; and “arm 5” which consists of only two helices. The hXRCC1 NLS peptide/Impα complex reveals a bipartite NLS in the structure in which residues from both ends of the peptide sequence, G^244^KRKL and S^268^VPKRPKLP, occupy the minor and major recognition sites of Impα centered around arm repeats 6–7 and 2–4, respectively (Fig. 2a). A stereo image of a portion of the electron density map showing the major motif in the Impα major binding pocket is shown in Supplementary Fig. S1. The major site structure includes 14 hydrogen bonds below the 3.3 Å cutoff, while the minor site includes 10 (Fig. 2b,c). The more extensive interactions with the nonapeptide are typical for major pocket binders, and include non-specific interactions with the carbonyl oxygens of the SVP residues preceding the basic consensus sequence: K-(K/R)-X-(K/R). The observed interacting segments are generally similar to those seen for the yeast cap binding protein 80 (CBP80; pdb: 3UKY)^31^, and this similarity is reflected by the close structural agreement of the two bound segments (RMSD = 0.28). The XRCC1 NLS peptide/Impα(70–529) structure provides direct structural support for an extended NLS sequence^15^ and is consistent with the nuclear localization data described below. Interestingly, the two bound regions correspond to the two most probable monopartite sequences identified by the program NLS Mapper^16^. We note finally that a comparison of the sequences of human and murine Impα(70–529) indicates that none of the residues that differ between the two species makes contact with the hXRCC1 NLS peptide ligand (Supplementary Fig. S2).

### Functional requirement for the NLS motifs

In order to relate the structural data summarized above and the functional contributions of the two motifs in the bipartite NLS, we evaluated the nuclear localization of a green fluorescent protein (GFP)-wild-type XRCC1 fusion protein and similar GFP fusions lacking either the intact minor motif (M1), major motif (M2), or both peptide motifs. The wild-type fusion showed a high degree of nuclear localization with almost no cytoplasmic staining (Fig. 3a, columns 1 and 2). We used a whole cell stain HCS CellMask™ Red Stain to accurately assess the cytoplasmic contribution and determine the entire cytoplasmic area (Fig. 3a, column 3). The nuclear to cytoplasmic (N/C) ratio for the wild-type fusion protein was 9.33 ± 0.64 (Fig. 3b). The ΔM1 construct showed a significant reduction in nuclear localization, with the N/C ratio falling to 2.86 ± 0.17, while a larger reduction was observed for the ΔM2 construct (N/C = 1.26 ± 0.08). The ΔM1 & ΔM2 construct (N/C ratio = 1.02 ± 0.06) showed the strongest defect in nuclear localization with p values < 0.01 compared to wild-type and ΔM1 and a p value of 0.025 compared to ΔM2. These results indicate that both motifs of the observed bipartite NLS contribute to efficient nuclear import.

### NLS peptide binding studies

**Figure 1:**
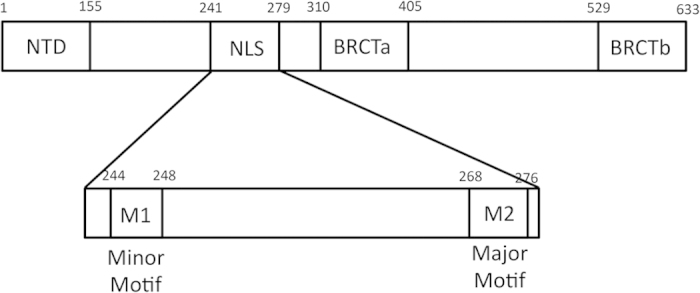
Domain structure of XRCC1 and position of the XRCC1 bipartite NLS. The figure also identifies the positions of the minor (M1) and major (M2) binding motifs.

**Figure 2:**
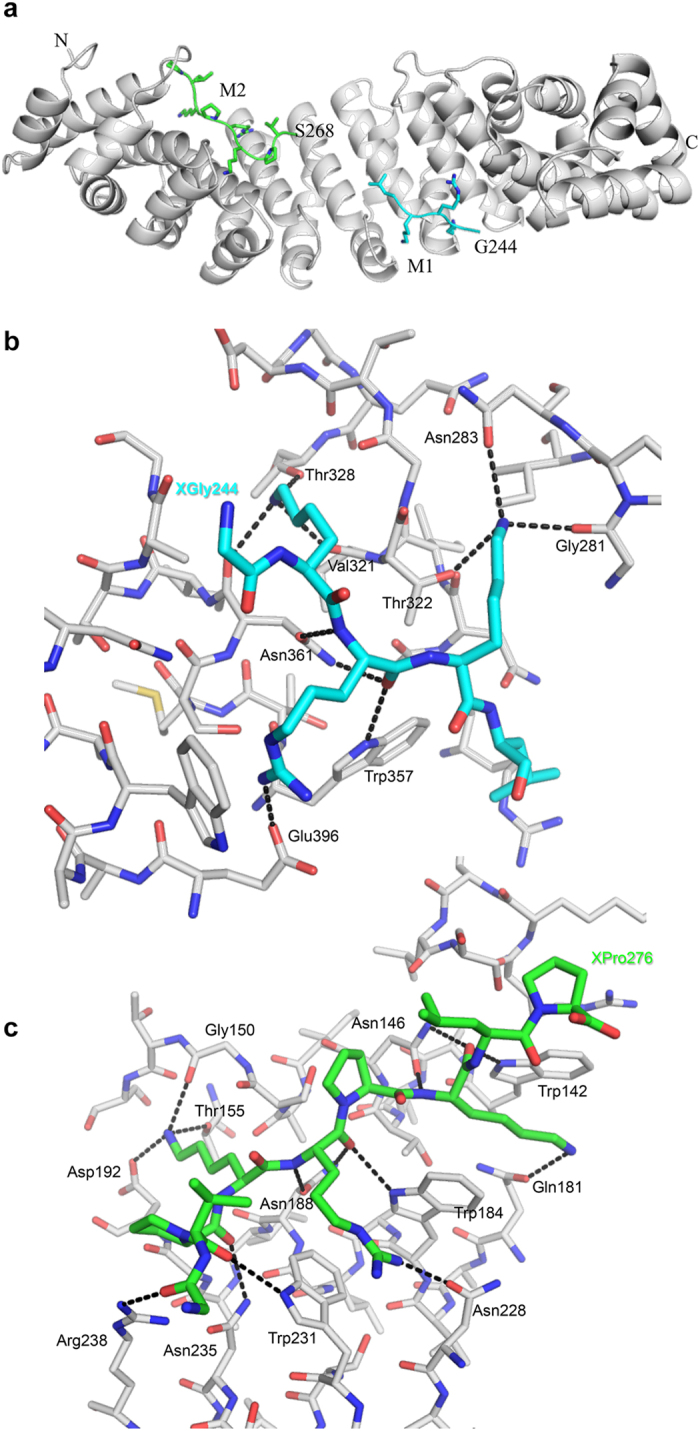
Crystallographic structure of Importin α-NLS peptide complex. (**a**) Ribbon diagram showing the Impα(70–529) complex with the XRCC1 NLS peptide. The NLS minor motif is in cyan and the major motif is in green; (**b**) Expanded region showing the Impα(70–529)—minor motif ligand complex; (**c**) Expanded region showing the Impα(70–529) major motif ligand complex. Linker residues from 249–267 are not observed due to disorder.

**Figure 3:**
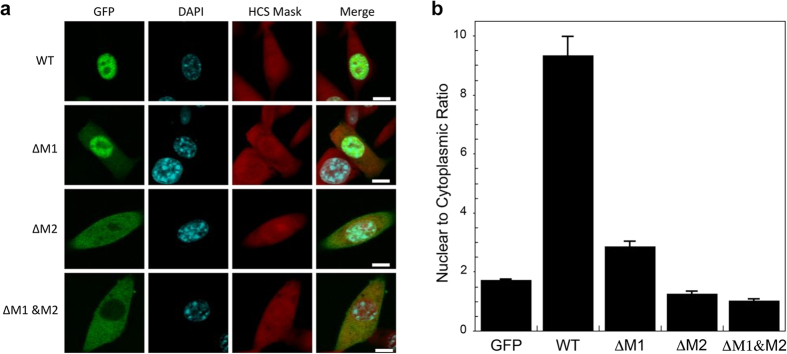
Localization of hXRCC1-GFP NLS mutants. (**a**) The GFP column shows the cellular distribution of the hXRCC1-GFP fusion for wild-type XRCC1, and for analogs lacking either the minor motif (M1), the major motif (M2) or both. The DAPI column (DNA staining) defines the cell nucleus. The HCS mask column (whole cell staining) defines the entire cell area. The Merge column superimposes the GFP, nuclear stain, and whole cell stain to reveal the extent of nuclear localization for each XRCC1 construct. Scale bars are 10 μm; (**b**) Bar graph showing the effect on nuclear localization of deletions of the minor motif (ΔM1), the major motif (ΔM2) or both (ΔM1 & ΔM2). The ΔM1 and ΔM2 constructs and the fluorescence methodology are described in Methods.

**Figure 4:**
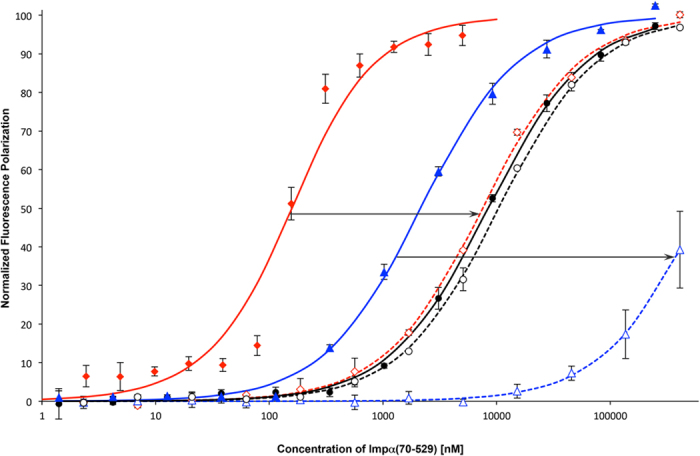
Binding curves for XRCC1 NLS peptide constructs. Solid lines with filled data markers show normalized fluorescence polarization as a function of the concentration of Impα(70–529) and dashed lines with open data markers show normalized fluorescence polarization as a function of the concentration of the variant Impα(70–529)(W184R/W231R). The FITC-labeled bipartite XRCC1 NLS (red), and the FITC-labeled minor motif (black) and major motif (blue) peptides are defined in Table 1. Titrations were performed at 25° C. The horizontal arrows illustrate the affinity changes that obtain when the major binding pocket is blocked by mutation. The data points were fit to a single-site non-cooperative model described in Methods. Error bars show the standard deviation of duplicates.

We determined equilibrium dissociation constants for the interaction of XRCC1 NLS peptide constructs with Impα(70–529) using fluorescence polarization anisotropy experiments with fluorescein isothiocyanate (FITC)-labeled peptides corresponding to the major motif (M2), the minor motif (M1), and the full bipartite NLS. In these studies, the major and minor motifs were defined based on the observation of residues in the crystal structure of the NLS peptide- Impα(70–529) complex. In both cases, the sequences extend beyond the consensus sequence (Fig. 1). For example, the major motif extends beyond the KRPK consensus sequence on both ends, i.e., SVPKRPKLP. Titration curves obtained using the FITC-labeled peptides are shown in Fig. 4, and yielded K_d_ values of 8.4 μM (M1), 2.1 μM (M2), and 0.10 μM (bipartite NLS) (Table 1). It also is apparent from Fig. 4 that, in contrast with the other examples studied, binding of the bipartite XRCC1 NLS peptide is not accurately described using a standard, single-site binding formalism.

In order to further investigate the interaction of the NLS motifs with the Impα major and minor binding pockets, we prepared a variant of Impα(70–529) containing the major site mutations W184R and W231R. These two modifications replace tryptophan residues in the Impα major binding pocket that play prominent roles in NLS binding, with arginine residues that unfavorably interact with typical cationic binding peptides (Supplementary Fig. S3). Consistent with expectations (Fig. 4), the titration studies using the mutant Impα(70–529)(W184R/W231R) revealed that: 1) the interaction of the bipartite NLS peptide exhibits significantly reduced binding affinity, with a K_d_ value similar to that of the minor (M1) binding motif peptide; 2) the binding behavior of the minor binding motif peptide is essentially unaffected; and 3) the major binding motif (M2) peptide interacts only weakly, having an apparent K_d_ ~ 600 mM. This extremely weak affinity of the major motif peptide demonstrates that there is substantial binding specificity, as the peptide clearly does not interact well with the unperturbed minor binding pocket in Impα(70–529)(W184R/W231R). Most importantly, these results support the roles of the specific interactions of the bipartite XRCC1 NLS motifs with both the major and minor binding pockets and further indicate that non-specific binding interactions do not contribute significantly to the affinity of the bipartite NLS for Impα(70–529).

The sequence of the human XRCC1(241–276) NLS contains TPS and SPS motifs in the region between the minor and major binding motifs, which are potential sites for phosphorylation. We obtained an analog of our NLS peptide that includes four glutamate substitutions: T257E/S259E/S266E/S268E, designed to mimic phosphorylation of the serine and threonine sidechains, and studied its binding to Impα(70–529) using the fluorescence polarization competition assay described in the Methods section. This peptide exhibits weaker interaction with Impα(70–529) as the K_d_ is nearly 6 fold higher (Table 1) than the wild-type sequence, indicating that phosphorylation of these residues may be involved in regulating XRCC1 NLS binding to Impα.

### Cooperative binding model

**Figure 5:**
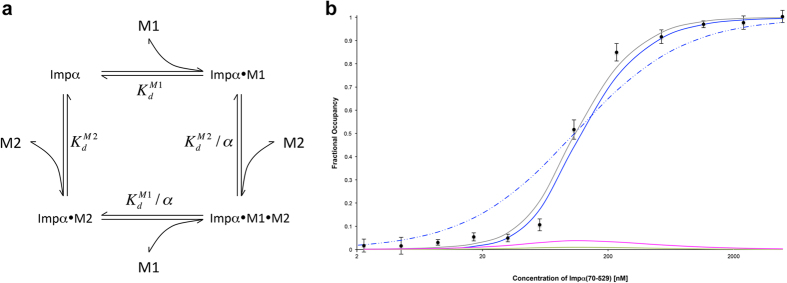
Cooperative binding model. (**a**) In the kinetic scheme for cooperative binding, 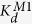 refers to the dissociation constant for the interaction of Impα(70–529) with the minor motif peptide and 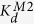 refers to the dissociation constant for the interaction of Impα(70–529) with the major motif peptide; (**b**) For curve-fitting to the cooperative binding model, titration data for the FITC-labeled bipartite NLS were converted to fractional occupancy and fit using the cooperative binding model of Fig. 5a (gray line). A simulated binding curve using the single site model of equation [2] with K_REF_ = 0.108 uM (broken blue line), and the simulated curves corresponding to ρ_M1M2_ (solid blue line), ρ_M2_ (magenta line), and ρ_M1_ (green line) from equation [1] are also shown.

The studies presented above demonstrate that the bipartite XRCC1 NLS peptide exhibits a binding affinity significantly greater than that of the major or minor motif peptides, and further that non-specific binding interactions do not play a role in explaining this difference. Here, we consider an alternative way to view the interaction of the bipartite NLS peptide with Impα. Our view assumes a cooperative binding model for the interactions of the major and minor NLS motifs with Impα. The cooperativity is proposed to result from the tethering of the two binding motifs, such that after the initial binding of either motif, the increased local concentration of the second binding motif near its cognate site results in a greatly accelerated association rate. This mechanism differs from the more commonly encountered instances of cooperative binding involving non-linked molecules that results from protein-mediated conformational changes^32^ or ligand-ligand interactions. For the example of NLS binding, the effect is not conformationally mediated, since Impα appears to be a reasonably rigid structure. Instead, the binding enhancement results from an effect on the binding kinetics that is a consequence of spatial localization. In the scheme shown in Fig. 5a, binding motifs M1 and M2 exhibit different dissociation constants from their respective sites, but there is a common cooperativity parameter α. The fractional occupancy of sites for M1 or M2 or both M1 and M2 simultaneously is given by the expressions^33^:


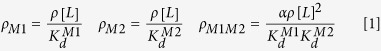


where:


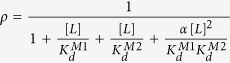


and where: 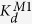 is the dissociation constant for the interaction of the minor motif with the minor binding pocket; 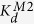 is the dissociation constant for the interaction of the major motif with the major binding pocket; and α is the cooperativity parameter.

Successful nuclear import is presumed to result primarily from an NLS- Impα complex with both the M1 and the M2 sites occupied, and thus is determined by ρ_M1M2_. The corresponding fractional occupancy can be compared with that predicted for a monopartite NLS peptide with dissociation constant K_REF_:


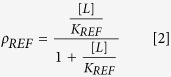


As noted above and illustrated in Fig. 4, most of the binding curves measured are adequately described by a simple binding model, while binding of the bipartite NLS peptide produces a much steeper response that is not well approximated by a single site binding model. In Fig. 5b, the normalized fluorescence polarization data for binding of the bipartite NLS peptide were fit using either the simple binding model of equation [2] or the cooperative binding model of equation [1] (see Supplementary Methods), setting 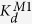 = 8.4 μM and 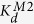 = 2.1 μM while optimizing the cooperativity parameter α. The gray curve shown in Fig. 5b corresponds to the sum of ρ_M1_, ρ_M2_, and ρ_M1M2_; this leads to α = 633 ± 38, with a half-saturation point of 0.116 ± 0.002 μM, i.e., nearly equivalent to the apparent K_d_ value obtained using the simple fit (Table 1, Fig. 5b, broken blue curve). Low fractions of the two singly occupied species, ρ_M1_ and ρ_M2_, are also predicted by the model. Despite the low levels of the single-motif bound Impα-NLS complexes, these complexes can potentially play an important role in cargo protein unloading. We note as well that the cooperative binding model predicts a much greater degree of Impα saturation for concentrations above the K_d_ value, allowing transport to become more efficient.

## Discussion

The function of DNA repair enzymes is contingent on their localization to the nucleus or other DNA-containing compartments^34^,^35^. The central role of scaffold proteins that bind these repair enzymes and orchestrate the repair process is increasingly recognized^36^,^37^,^38^,^39^. In the case of XRCC1, a scaffold protein of primary importance in the SSBR and BER pathways, nuclear translocation also includes its binding partners Lig3α, which lacks an NLS and therefore depends on XRCC1 for co-transport, JWA^29^, and possibly other repair enzymes. The present study provides structural and functional cellular data that resolve earlier inconsistencies regarding the identity of the XRCC1 bipartite NLS^8^,^15^. Computational NLS prediction software^16^,^40^,^41^ provided useful preliminary identifications, but failed to identify the full bipartite NLS, presumably because the algorithm has difficulties when separation of the major and minor binding motifs is too great.

Phosphorylation by casein kinase 2 (CK2) has been shown to promote nuclear accumulation of XRCC1^42^. Phosphorylation of the T^257^PS and S^266^PS motifs in the XRCC1 NLS linker, modeled by an NLS(T257E/S259E/S266E/S268E) quadruple variant peptide (NLS-E4), resulted in reduced binding affinity for Impα(70–529) (Table 1), however, these residues are not consensus sites for CK2 phosphorylation. Studies of Parsons *et al.*^43^ have indicated that the effect of phosphorylation on nuclear localization results primarily from antagonizing XRCC1 ubiquitylation and degradation, raising the levels of XRCC1 in both the cytosol and the nucleus. Thus, for the case of XRCC1, the basis of phosphorylation-dependent nuclear accumulation appears not to be directly related to the nuclear transport system.

**Figure 6:**
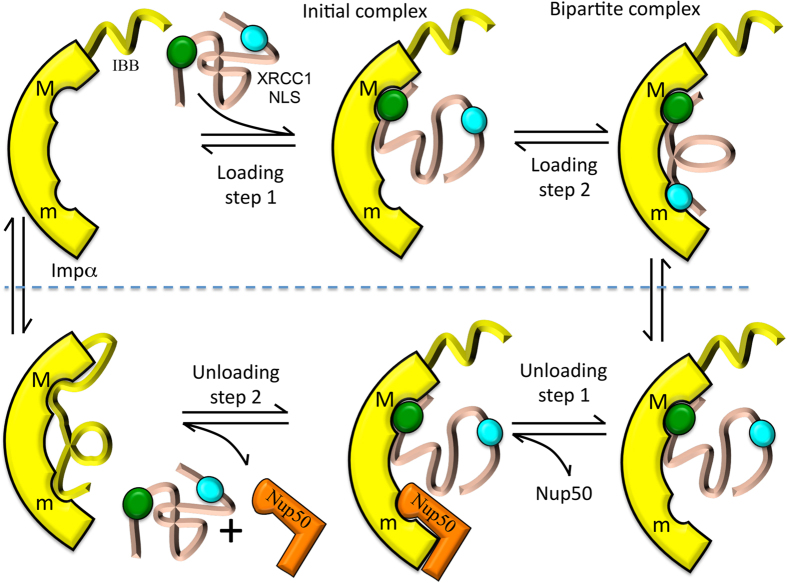
Stepwise loading and unloading of a bipartite NLS peptide. The upper panel illustrates the stepwise loading of Impα with a bipartite NLS, and the lower panel illustrates the stepwise unloading. The major (minor) motif binding site is labeled M (m). Loading can involve an initial binding of either the major or minor motif, while unloading will typically involve initial dissociation of the lower affinity minor motif and its replacement by Nup50 or IBB following Importin β dissociation.

An efficient nuclear transport system requires both sufficient binding affinity for the translocation of large and complex molecules and rapid loading/unloading behavior—characteristics that are fundamentally in opposition. The cooperative binding behavior of the bipartite NLS provides one solution to these inconsistent requirements. Cooperative binding behavior results in a ligand with much greater binding affinity than that of either of its separate binding motifs. However, the small fractions of partially bound species provide an opportunity for competitive binding by unloading factors such as Nucleoporin 50 (Nup50) (Supplementary Fig. S4) and the Impα IBB peptide^44^,^45^. Once the minor motif binding pocket on Impα becomes blocked by IBB or Nup50, the remaining major site complex dissociates without the cooperative contribution of minor site binding (Fig. 6).

For our *in vitro* crystallization and binding studies we used the mouse Importin α2 IBB-deletion construct, Impα(70–529), because it has been well characterized in previous studies, e.g.^17^,^30^,^31^,^44^,^45^,^46^,^47^. Human Importin α exists in seven different isoforms^48^ that are grouped into 3 families. It has been shown that NLS peptides bind to all the isoforms with very similar affinities *in vitro*^49^ even though there is selectivity of isoforms for particular cargo proteins^50^. Our experiments do not address specificity of isoforms for XRCC1 nuclear transport; however, based on available structural data, the binding characteristics of the XRCC1 NLS are likely to be similar for the different isoforms.

In the studies of XRCC1 NLS binding described here, the cooperative binding behavior is demonstrated both by a comparison of the apparent binding constants of the individual binding motifs and the complete bipartite NLS, and by the shape of the binding curves. The origin of this cooperativity differs from that of non-linked ligands where cooperative binding generally results from protein-mediated conformational changes or direct ligand-ligand interactions. For bipartite NLS ligands, cooperativity results from linker-constrained localization leading to an increased association rate for the second binding step. The cooperativity parameter obtained for the XRCC1 NLS motifs is larger than that typically found for conformationally mediated effects, although very large cooperativity parameters have occasionally been reported for allosteric proteins as well^51^. The binding studies complement the cell imaging data indicating the cooperative contributions of the major and minor motifs on nuclear localization of XRCC1, as well as previous phenomenological studies demonstrating cooperative localization contributions of major and minor motifs in other systems^52^,^53^,^54^,^55^.

Three mechanisms by which the bipartite NLS linker can influence NLS binding cooperativity are: 1) by influencing the separation of the major and minor motifs in the conformational ensemble to be similar to the separation of the cognate binding sites; 2) by influencing the conformation of the major and minor NLS motifs; and 3) by forming direct interactions, e.g., salt bridges, with residues involved in Impα binding. Some effects on relative positioning have been investigated by Lange *et al.*^19^ who determined that poly(alanine) linkers longer than 13 residues were non-functional. The importance of an extended conformation for the major motif is indicated by the presence of proline residues before, after, and within the XRCC1 NLS major motif (Table 1). These residues eliminate helical conformations while favoring the extended conformation that is observed in the Impα complex. Alternatively, interactions that promote helix formation^56^ or form competing H-bonds with the backbone amides can reduce binding by reducing the association rate of the binding motif in an incorrect conformation. Formation of salt bridges that compete with those in the NLS-Impα complex provides one possible explanation for the reduced binding constant observed for the NLS-E4 analog (Table 1).

As recently discussed by Wirthmueller *et al.*^57^, the NLS affinity required for efficient Impα-mediated nuclear uptake has been controversial. Comparison of reported binding studies indicates that some of this variability appears to be related to the methodologies used, and in particular with the nature of the test NLS peptide. Another potential source of error, demonstrated by the studies described above, may arise from treating a cooperative binding curve with a formalism that assumes only a single binding interaction. Effects of the linker residues have frequently been attributed to non-specific binding contributions (e.g.^58^). In the studies reported here, binding of the full NLS peptide to the major pocket-blocked Impα variant resulted in affinity similar to that of the minor (M1) motif, thus eliminating the possibility of significant contributions by NLS peptide residues not included in this motif. In general, it is difficult to attribute large binding contributions to residues that interact too weakly to show up in crystallographic characterizations. Although non-specific binding interactions are undoubtedly significant in some cases, these results indicate that a primary role of the linker is to produce cooperative binding rather than to interact directly with Impα.

## Methods

### Protein Production

A His-tagged murine Importin α protein with the Importin β binding domain (IBB) deleted, which is identical to Impα(70–529) studied by Fontes *et al.*^30^, was expressed in *E. coli*. The coding sequence was purchased from Genscript and cloned into the pET30 expression vector between BamHI and SalI restriction sites to yield the his-tagged construct. The major binding pocket double mutant W184R/W231R was created using a Quikchange site-directed mutagenesis kit (Agilent). All clones were sequence verified.

BL21(DE3) transformants were grown at 37 °C in LB broth to A_600_ = 1 and then isopropyl β-D-1-thiogalactopyranoside was added to a concentration of 1 mM and the cultures were incubated at 18 °C overnight with shaking. Induced cells were harvested by centrifugation, resuspended in binding buffer (20 mM sodium phosphate, 500 mM NaCl, 1 mM MgCl_2_, 25 mM imidazole, 1 mM DTT), lysed by sonication, and the protein was purified by nickel affinity chromatography using a HisTrap column (GE Healthcare). The column was eluted with buffer containing 500 mM imidazole, and the protein was further purified by gel filtration on a HiLoad 26/60 Superdex 200 column (GE Healthcare) with an elution buffer containing 20 mM Tris, pH 7.8, 125 mM NaCl, 1 mM EDTA, and 2 mM DTT. Protein yield typically exceeded 200 mg per liter of culture. The expression behavior of Impα(70–529)(W184R/W231R) was essentially identical to that of the wild-type and the protein was purified following the same protocol. Protein concentrations were determined by the Edelhoch procedure^59^.

### Crystallography

Crystals of murine Impα(70–529) in the presence of N-acetylated hXRCC1(241–276) peptide (Genscript), designed to include the putative major and minor motifs, were grown at 20 °C using sitting drop vapor diffusion. The sample was prepared by mixing 8.8 mg/ml of Impα(70–529) with 2.8 mg/ml of the peptide (4.4-fold excess) in 125 mM NaCl, 20 mM Tris-HCl, pH 7.8, and 5 mM DTT. 1 μL of protein solution was added to 2 μL of reservoir solution containing 1.5 M ammonium sulfate, 0.1 M bis-tris propane, protease inhibitor cocktail (Amresco), pH 7.0. For data collection, crystals were transferred to a cryoprotective solution containing reservoir buffer plus 20% glycerol and then flash frozen in liquid nitrogen

A low-resolution data set of the Impα(70–529)-XRCC1 peptide complex was collected at 2.6 Å resolution on an in-house 007HF Rigaku generator equipped with a Saturn92 CCD detector. Data were processed using HKL2000 and the molecular replacement was solved using PHASER^60^ with Importin coordinates from 3UKY (pdb idcode) as the starting model followed by subsequent partial refinement in Phenix^61^. A higher resolution data set was collected at 2.3 Å at the Southeast Regional Collaborative Access Team (SER-CAT) 22-ID beamline at the Advanced Photo Source, Argonne National Laboratory. The data were processed in HKL2000 and refined in Phenix and COOT using the model from the low-resolution data as the starting structure. The final structure displayed good geometry with 100% of the residues in the allowed region of the Ramachandran plot. The same Rfree flags were maintained for these two data sets. Table 2 shows the structure statistics.

### Peptide binding assays

Apparent peptide dissociation constants were determined based on fluorescence polarization measurements using FITC-labeled peptides. Impα(70–529) was exchanged into FP buffer (20 mM Tris, pH 7.7, 125 mM NaCl, 1 mM EDTA, 2 mM *tris*(2-carboxyethyl)phosphine, 5% sucrose, 10 mM NaN_3_). Titrations of FITC-labeled peptides were performed using serial dilutions of Impα(70–529) in a mixture with a final concentration of 100 nM FITC-labeled peptide in FP buffer. 50 μL volumes of the binding reactions were pipetted into wells of a 96-well black flat bottomed plate and fluorescence polarization was read at room temperature with the POLARstar Omega microplate reader (BMG Labtech) using excitation at 485 nm and emission at 520 nm. Impα(70–529) binding experiments were done in duplicate and repeated with different preparations of protein. Impα(70–529)(W184R/W231R) binding experiments were done in duplicate. The fluorescence polarization values generated by the experiments are arbitrary, so for easy comparisons among samples, the data were normalized to a 0–100 scale after curve-fitting. Data were fit to a single-site binding equation for the interaction of a peptide (L) with Impα (M) as described below:


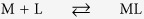



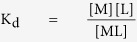



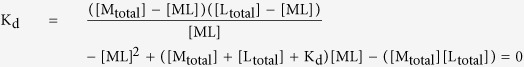


Solve for [ML] using the quadratic formula:


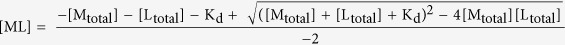


Define the fraction bound = *θ*:


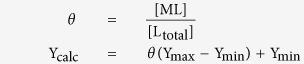


Where Y_calc_ represents the fluorescence polarization value that is generated by the model. The nonlinear curve-fitting algorithm in the Microsoft Excel Solver was used to fit the experimentally determined fluorescence polarization values (Y_obs_) to Y_calc_ using fitting parameters Y_max_ (the upper limit), Y_min_ (the lower limit), and the K_d_.

Fluorescein-labeled peptides (Genscript) used were: Minor motif ligand (M1): FITC-GG-XRCC1(244–248); Major motif ligand (M2): XRCC1(268–276)-G-Lys-FITC; Full XRCC1 NLS ligand (FL-NLS): FITC-XRCC1(241–276). The FITC tags were selected in an attempt to minimize possible non-specific interactions of the fluorescein with the Impα(70–529). Concentrations of FITC-labeled peptides were determined by absorbance at 492 nm using an extinction coefficient of 75,000^ ^M^−1^cm^−1^. Concentrations of unlabeled peptides were determined by UV absorbance^62^.

The binding constant of the unlabeled peptide (NLS-E4): XRCC1(241–276)/(T257E/S259E/S266E/S268E) quadruple variant was determined using a fluorescence polarization competition assay. Titrations were performed by using serial dilutions of unlabeled peptide in a mixture with fixed concentrations of Impα(70–529) (150 nM) and FITC-labeled FL-NLS peptide (100 nM) in FP buffer. 50 μL volumes of the binding reactions were pipetted into wells of a 96-well plate, and fluorescence polarization was determined as above. Experiments were done in duplicate and repeated with different preparations of protein. Data were fit to a competitive binding equation^63^ using the same three parameter fit software described above.

### Cell lines and plasmids

Xrcc1^−/−^ p53-deficient mouse embryonic fibroblasts were obtained from Dr. Robert Tebbs^64^. These cells were maintained in low glucose Dulbecco’s Modified Eagle’s medium (Invitrogen, Carlsbad, CA) supplemented with 10% fetal bovine serum (FBS) (HyClone, Logan, UT) in a 10% CO_2_ incubator at 37 °C. Mycoplasma testing was performed routinely using a MycoAlert^®^ Mycoplasma detection kit (Lonza, Rockland, ME) and results were negative.

Human XRCC1 with a C-terminal GFP (RG204952, pCMV-AC-GFP) was purchased from Origene (Rockville, MD). Site-directed mutagenesis was utilized to create XRCC1-GFP variants lacking residues 245–247 (ΔM1), lacking residues 271–274 (ΔM2), or lacking residues 245–247 and residues 271–274 (ΔM1 & ΔM2). Mutants were created using a Quikchange site-directed mutagenesis kit (Agilent). All clones were sequence verified.

### Fluorescence Microscopy

Cells were seeded in 35 mm glass bottomed petri dishes (MatTek, Ashland, MA) at 2 × 10^5^ cells per dish and incubated in growth medium for 24 h. After 24 h, cells were transiently transfected with the indicated GFP-fusion protein using Lipofectamine 2000 (Life Technologies, Carlsbad, CA). 24 h after transfection, cells were fixed with a 3.7% neutral buffered formaldehyde solution (Thermo Scientific) for 1  min at room temperature. Cells were then washed three times with phosphate-buffered saline (PBS, Hyclone). After fixation, cells were permeabilized with 0.25% Triton X-100 in PBS for 10 min, washed three times in PBS, then the whole cells were stained with HCS CellMask™ Red Stain per manufacturer’s protocol (Life Technologies). Cells were then washed three times with PBS, and nuclei were stained with NucBlue® Fixed Cell Stain ReadyProbes™ (Life Technologies) for 5 min. Fluorescence images were acquired with a 40 × C-Apochromat (numerical aperture 1.2) water immersion objective coupled to a Zeiss LSM510 META confocal microscope (Carl Zeiss MicroImaging). Multi-track configuration was used to ensure the absence of excitation cross-talk or emission bleed-through between channels. The 364 nm laser line was used at 3.5% maximum intensity, the 488 nm laser line was used at 10% of maximum intensity and the 543 nm laser line was used at 100% of maximum intensity. For GFP imaging, the 488 nm laser line with a 505–550 bandpass filter was used with a gain setting of 580–610 for all quantitative imaging acquisition. Gain setting was determined by examining transiently transfected cells and creating a threshold range that best recapitulated immunofluorescent staining of endogenous XRCC1. HCS CellMask™ Red imaging was done using the 543 nm laser line with a 560–615 bandpass. 4′,6-diamidino-2-phenylindole (DAPI) imaging was with the 364 nm laser line and 385–470 bandpass filter. 2-D images were acquired and DAPI staining was used to select the best focal plane for nuclear imaging. Images were acquired with a pinhole of 1 airy unit (AU), a zoom of 1.0. Zen 2009 software was used for all image acquisition.

### Fluorescence Intensity Analysis

Images were analyzed using MetaMorph. Nuclear and cytoplasmic boundaries were determined using the images taken from the DAPI and HCS Cell Mask™ channels, respectively. The intensity of the GFP for each of these regions was determined from the GFP channel. The intensity indicates the relative amount of XRCC1 that is localized to either cellular compartment. The ratio of the nuclear intensity of GFP to the cytoplasmic intensity of GFP was taken to represent the extent of nuclear localization.

Forty to sixty cells were analyzed in this manner for each fusion protein. Accumulation of free GFP in the nucleus of Xrcc1^−/−^ cells was measured as a control (N/C = 1.7 ± 0.05). N/C values of all cells for each construct were averaged to determine a mean value. Values that were 2 standard deviations above or below the mean value were determined to be outliers and were eliminated.

## Additional Information

**How to cite this article**: Kirby, T. W. *et al.* Nuclear Localization of the DNA Repair Scaffold XRCC1: Uncovering the Functional Role of a Bipartite NLS. *Sci. Rep.* **5**, 13405; doi: 10.1038/srep13405 (2015).

## Supplementary Material

Supplementary Information

## Figures and Tables

**Table 1:** Apparent ligand Dissociation Constants.

Peptide	Sequence	Impα(70–529) K_d_ (μM)^a^	W184R/W231R K_d_ (μM)^b^
FL-NLS: FITC-XRCC1 (241–276)	FITC^c^-SPKGKRKLDLNQEEKKTPSKPPAQLSPSVPKRPKLP	0.108 ± 0.007	7.4 ± 0.3
M2: XRCC1 (268-276)-G-Lys-FITC	SVPKRPKLP-G-LYS-FITC	2.1 ± 0.08	690 ± 280
M1: FITC-GG-XRCC1 (244-248)	FITC-GGKRKL	8.4 ± 0.6	10.4 ± 0.7
NLS-E4	SPKGKRKLDLNQEEKKEPEKPPAQLEPEVPKRPKLP	0.604 ± 0.061	—

^a^K_d_ ± Standard error of mean.

^b^K_d_ ± Standard deviation.

^c^FITC = Fluorescein isothiocyanate.

**Table 2:** Data collection and refinement statistics.

Data collection	
Space group	P2_1_2_1_2_1_
Cell dimensions	
*a*, *b*, *c* (Å)	78.67, 90.11, 100.70
α, β, γ (°)	90, 90, 90
Resolution (Å)	50.0 (2.3)^*^
*R*_sym_ or *R*_merge_	0.08 (0.35)
*I*/σ*I*	9.6 (2.3)
Completeness (%)	96.6 (83.2)
Redundancy	5.4 (3.0)
Refinement	
Resolution (Å)	50.0 (2.3)
No. reflections	56,836
*R*_work_/*R*_free_	17.4/20.8
No. atoms	
Protein (Importin)	3152
Ligand (XRCC1 peptide)	111
Solvent	241
*B*-factors	
Protein (Importin)	38.1
Ligand (XRCC1 peptide)	45.7
Solvent	42.8
R.m.s. deviations	
Bond lengths (Å)	0.002
Bond angles (°)	0.680

*Values in parentheses are for highest-resolution shell.
