# Supplementary material for: Nuclear Localization of the DNA Repair Scaffold XRCC1: Uncovering the Functional Role of a Bipartite NLS
Source: Sci Rep. 2015 Aug 25;5:13405. doi: 10.1038/srep13405 (PMC4548243; doi:10.1038/srep13405)
Supplement: Supplementary Information [file srep13405-s1.pdf]

Supplementary Figures

Nuclear Localization of the DNA Repair Scaffold XRCC1: Uncovering the Functional Role of a Bipartite NLS

Thomas W. Kirby, Natalie R. Gassman, Cassandra E. Smith, Lars C. Pedersen, Scott A. Gabel, Mack Sobhany, Samuel H. Wilson, Robert E. London

Supplementary Figure S1.

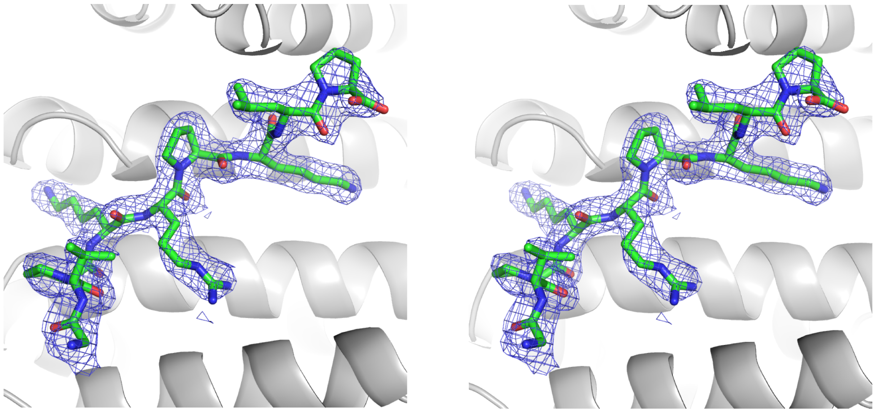

Representative electron density map of XRCC1 NLS peptide/Impα(70-529). The stereo view of the M2 site of a *2Fo-Fc* electron density map contoured at 1.0 σ is shown.

Supplementary Figure S2.

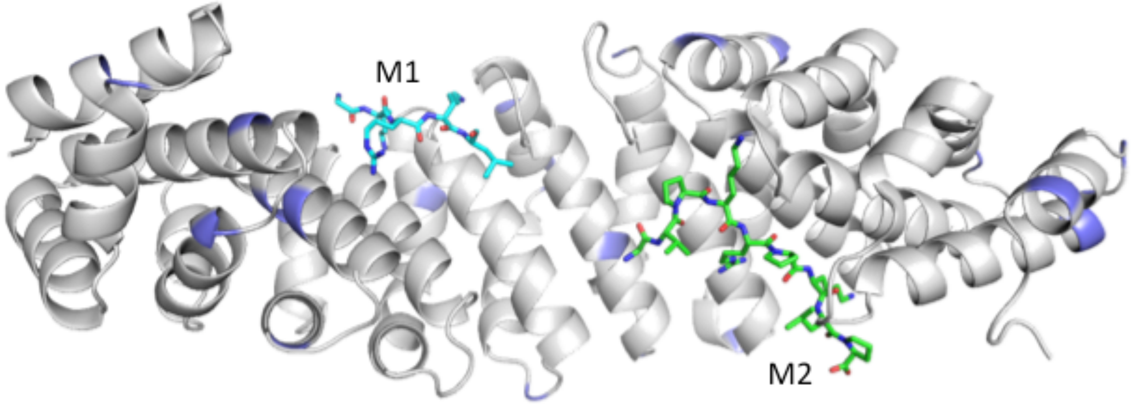

Positions of residues that differ in the human and mouse Impα(70-529). The figure shows a ribbon diagram of the complex formed by Impα(70-529) (gray) with the XRCC1 NLS (minor site motif, cyan; major site motif, green). Positions of residues that differ between the mouse and human forms are indicated in purple. Nearly all of the substitutions are conservative, and none of these residues makes direct contact with the XRCC1 NLS.

Supplementary Figure S3.

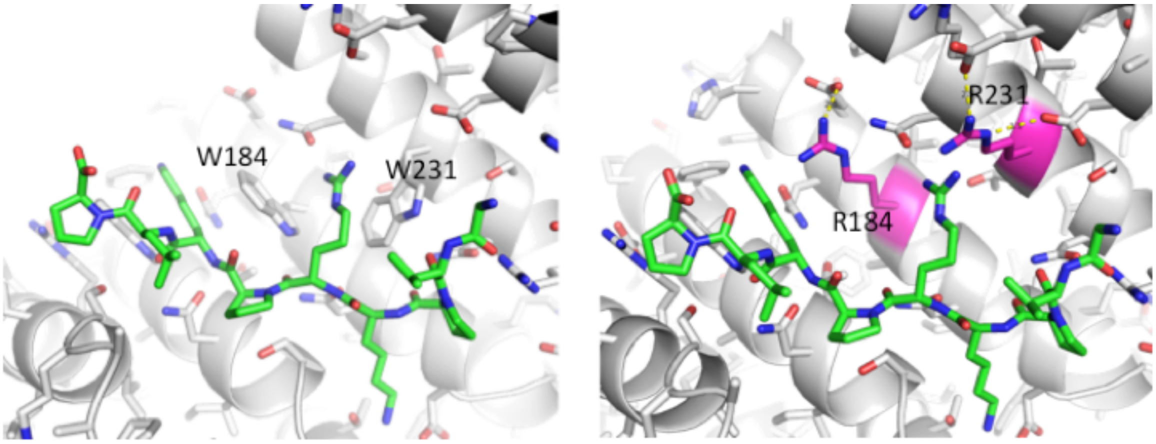

Mutational strategy used to block the Impα major site. The region of Impα involved in complex formation with the XRCC1 major site peptide (M2, green) is shown on the left, and the two mutations used to block the site are shown in magenta in the right panel. The double W184R, W231R mutations eliminate important binding interactions with M2, while introducing groups that are electrostatically and to some extent sterically incompatible with binding. The extended conformations of the Arg184 and Arg231 sidechains are predicted to be further stabilized by salt bridge interactions with Glu180 (Arg184) and with Glu266 and Asp270 (Arg284), so that no conformational change is expected.

Supplementary Figure S4.

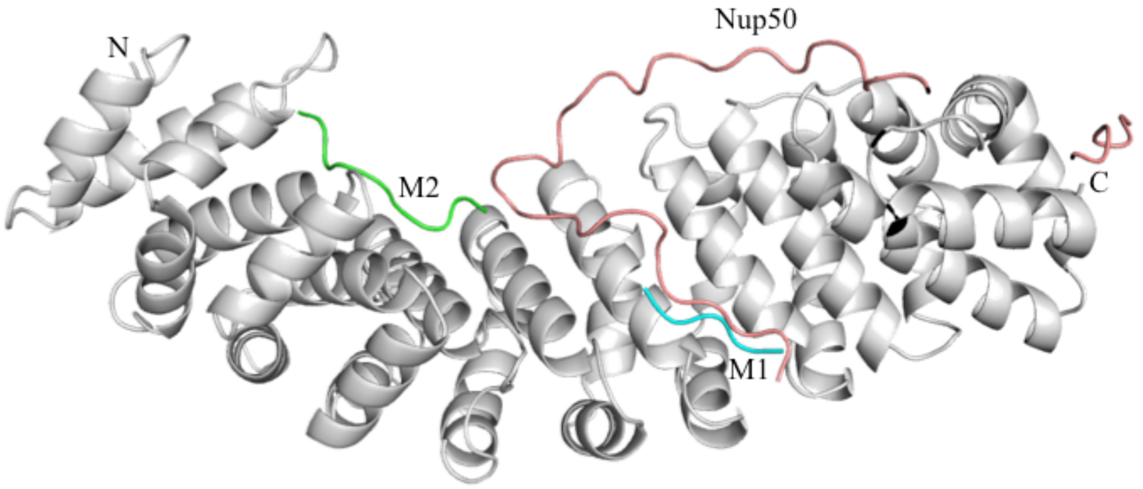

Comparison of the XRCC1 NLS and Nup50 binding to Impα. A ribbon diagram of Impα(70-529) with the XRCC1 NLS is overlaid with the diagram for the Nup50 complex (pdb: 2C1M, Matsuura and Stewart, 2005). The Nup50 can only form this complex when the minor site motif M1 is not present.

Supplementary Methods

For the cooperative model plot in Fig. 6, our titration experiment is performed by varying the concentration of Impα(70-529) at a fixed bipartite FITC-NLS concentration of 100 nM. In this case, the fractional occupancies in equation [1] correspond to the fractions of FITC-NLS that are bound to the major or minor (or both) binding pockets of Impα(70-529) and these protein sites can be thought of as the "ligand". As discussed by Gnacadja [33], the correct application of equation [1] requires use of the free rather than total ligand concentration; this distinction becomes significant at concentrations near the apparent K_d value. The free "ligand" concentration for each data point was determined by multiplying the sum of ρ_{M1}, ρ_{M2}, and ρ_{M1M2}, by the total concentration of FITC-NLS (100 nM) and then subtracting that result from the total concentration of Impα(70-529). The nonlinear curve-fitting algorithm in the Microsoft Excel Solver was used to fit the experimentally determined fluorescence polarization values using equation [1], using the fitting parameters Y_{max} (the upper limit), Y_{min} (the lower limit), and the cooperativity factor α. The K_d^{M1} and K_d^{M2} values were obtained from the separate fits of the FITC-labeled major and binding motif peptides (Table 1).
